# Mosaic trisomy 12 diagnosed in a female patient: clinical features, genetic analysis, and review of the literature

**DOI:** 10.1007/s12519-021-00438-9

**Published:** 2021-07-14

**Authors:** Daniela Hainz, Marcus Krüger, Daniela Reber, Karl Mehnert, Theresa Brunet, Gabriele Lederer, Sabine Langer-Freitag, Julia Hoefele

**Affiliations:** 1 Institute of Human Genetics, Klinikum rechts der Isar, School of Medicine, Technical University of Munich, Munich, Germany; 2 Department of Neonatology, Schwabing Hospital, School of Medicine, Technical University of Munich, Munich, Germany; 3 Genetikum, Genetic Counseling and Diagnostics, Stuttgart, Neu-Ulm, Germany

Mosaic trisomy 12 is a rare genetic condition with a highly variable phenotype. Clinical features associated with this condition include developmental delay, intellectual disability, dysmorphic facial features, short stature, pigmentary dysplasia, complex congenital heart defects and hypotonia (Table 1). To date, 20 patients have been described in which mosaic trisomy 12 was observed in both extraembryonic and neonatal/infant tissues. Chen et al. reported two cases without phenotypic abnormalities [1]. Of the 20 previously reported cases, 4 resulted in neonatal or infant death. Those findings support the hypothesis that mosaic trisomy 12 manifests across a wide spectrum of phenotypes and that predicting the degree of abnormalities is quite difficult [2–4]. In several cases, trisomy 12 mosaicism was detected prenatally in amnion fluid, but not postnatally [5–8]. Even if the mosaicism is not confirmed in one tissue, it may still be present in other tissues of the child.

**Table 1:** Reported cases of patients diagnosed postnatally with mosaic trisomy 12

Patient	Gender	Age at diagnosis	Tissue	Genetic testing	Karyotype (percentage of trisomic cells)	Clinical features at diagnosis	Clinical features at follow up	References
1	M	31 y	Peripheral blood	Chromosome analysis	47,XY,+12 (7%)	Kartagener syndrome with situs inversus, chronic sinusitis, bronchitis, and infertility	–	Richer et al. [19]
2	F	36 y	Skin fibroblasts	Chromosome analysis	46,XX	Severe intellectual disability, dysmorphic facial features, microcephaly, diffuse white matter disease, short stature, muscle stiffness, areflexia, limited extension of joints, waddling gaits and scoliosis, muscular hypotonia	–	Patil et al. [20]
			Peripheral blood	Chromosome analysis (2 analyses)	47,XX,+12 (13%)			
3	M	9 mon	Cord blood	Chromosome analysis	46,XX	Hypoglycemia, convulsion equivalents	Developmental delay (15 mon of age)	Leschot et al. [10]
			Skin fibroblasts	Chromosome analysis (2 analyses)	46,XX			English et al. [3]
			Urinary cells	Chromosome analysis (2 analyses)	47,XX,+ 12 (76.9–100%)		Normal mental development but “clumsiness” (6 y)	
4	F	1 mon	Peripheral blood	Chromosome analysis	46,XX	Dysmorphic craniofacial features, long and thin fingers, proximally placed thumbs, congenital heart defect	The child died from severe complications following surgical heart procedure at 5 wk of age	von Koskull et al. [14]
			Urinary cells	Chromosome analysis	46,XX			
			Skin fibroblasts	Chromosome analysis	47,XX,+12 (25%)			
5	F	Neonate	Peripheral blood	Chromosome analysis (2 analyses)	46,XX 47,XX,+12 (0.4%)	Dysmorphic facial features	Delayed motor development, normal intellectual development, short stature, scoliosis, pigmentary dysplasia, ASD, cardiomegaly (7 y)	English et al. [3]
			Skin fibroblasts	Chromosome analysis (2 analyses)	47,XX,+12 (9–13%)			
6	M	Neonate	Skin fibroblasts	Chromosome analysis	46,XY	Dysmorphic facial features, lung hypoplasia, Potter sequence	The child died one hour after delivery due to respiratory distress	Bischoff et al. [15] case B
			Spleen	FISH	47,XY,+12 (5%)			
7	F	9 mon	Peripheral blood	FISH	47,XX,+12 (4.2%)	Dysmorphic craniofacial features, asymmetric trunk and extremities	Normal growth, low-normal development (23 mon)	Aughton et al. [18]
			Skin fibroblasts	Chromosome analysis	47,XX,+12 (38%)/48,XX,+12,+20 (4%)			
8	F	10 mon	Cord blood	Chromosome analysis	45,X (82%)	Dysmorphic facial features, gross motor delays, linear hyperpigmented streaks on the inner calves and backs of the thighs, short neck, widely spaced nipples, hyperextensible joints	Supraventricular tachycardia, age appropriate cognitive development, gross motor delay, muscular hypotonia, hyperextensible joints, widely spaced nipples (18 mon)	Spiro et al. [17]
			Skin fibroblasts	Chromosome analysis	47,XX,+12 (15%)/45,X (45%)			
9	F	Neonate	Peripheral blood	Chromosome analysis	46,XX	Dysmorphic facial features, congenital heart defect, two-vessel cord, widely spaced nipples, camptodactyly, overlapping fingers, pigmentary dysplasia	The child died due to complications of the congenital heart defects at 2 mon	DeLozier-Blanchet et al. [12]
			Skin fibroblasts	Chromosome analysis	47,XX,+12 (15%)			
10	M	Neonate	Peripheral blood	Chromosome analysis	46,XX	Dysmorphic facial features, congenital heart defect, pituitary stalk interruption, polycystic ovary syndrome, strabismus, conductive hearing loss, muscular hypotonia, short stature	Delayed growth, normal cognitive development (15 y)	Boulard et al. [16]
			Ovarian fibroblasts	Chromosome analysis	47,XX,+12 (80%)			
11	F	Neonate	Cord blood	Chromosome analysis	47,XX,+12 (26%)	Dysmorphic facial features, cardiomyopathy (prenatal), edema	Neonatal death	Parasuraman et al. [21]
12	F	11 mon	Peripheral blood	Chromosome analysis	46,XX	Dysmorphic facial features, developmental delay, congenital heart defect, microcephaly, sensorineural hearing loss, tracheomalacia, intestinal malformation, pigmentary dysplasia, retinal pigmentary changes, mild muscular hypotonia	Intellectual disability, constipation, body asymmetry, mild muscular hypotonia (3 y)	Al-Hertani et al. [4]
			Skin fibroblasts	Chromosome analysis Hyperpigmented spots Hypopigmented spots	47,XX,+12 (19%) 46,XX			
13	F	6 mon	Cord blood	Chromosome analysis	46,XX	No phenotypic abnormalities	Normal in growth and psychomotor development (6 mon)	Chen et al. [1]
			Urinary cells	FISH	47,XX,+12 (5%)			
14	F	Neonate	Peripheral blood	Chromosome analysis	46,XX	Dysmorphic facial features, mild rhizomelic shortening of the limbs	Normal cognitive development, turricephaly, tall forehead, short neck, narrow palate, muscular hypotonia (6 mon)	Hong et al. [2]
				FISH	47,XX,+ 12 (6%)			
				Array	47,XX,+ 12 (25%)			
15	F	Neonate	Cord blood	Chromosome analysis	46,XX	No phenotypic abnormality	-	[22]
			Urinary cells	FISH	47,XX,+12 (7%)			
16	F	2 y	Peripheral blood	Chromosome analysis Chromosome analysis	46,XX 47,XX,+12 (28%)	Overgrowth	Neuropsychomotor developmental delay, prominent forehead, dolichocephaly, patchy skin pigmentation (2 y)	Gasparini et al. [23]
			Skin fibroblasts	FISH Hyperpigmented spots Hypopigmented spots	47,XX,+12 (36%) 47,XX,+12 (46%)			
17	F	2 mon	Peripheral blood	Chromosome analysis (3 analyses)	46,XX 46,XX 47,XX,+12 (6%) 46,XX	Dysmorphic facial features, anteriorly placed anus, pigmentary dysplasia, postaxial polydactyly of left hand, congenital heart defect, internal tibial torsion, mild left leg spasticity, tight heel cord, short stature	Developmental delay, intellectual disability (6 y)	Hu et al. [11] Case 1
				FISH				
			Skin fibroblasts	Chromosome analysis Array FISH	46,XX 46,XX 47,XX,+12 (5%)			
			Buccal cells					
18	F	Neonate	Peripheral blood	Chromosome analysis	46,XX	Premature, dysmorphic facial features, microcephaly, short stature, short neck, apnea, bradycardia, congenital heart defect, rectovestibular fistula, imperforate anus, cerebral atrophy, enlargement of the lateral ventricles, tethered cord, failure to thrive, chronic lung disease, bilateral conductive hearing loss, seizures, internal tibial torsion, minimal left extremity spasticity, tight heel cord	Developmental delay, intellectual disability, delayed growth (5 y)	Hu et al. [11] Case 2
				Array	47,XX,+12 (20%)			
19	M	Neonate	Peripheral blood	Chromosome analysis	46,XX	Premature, dysmorphic facial features, short stature, hyperopic astigmatism, mild tapered fingers, hypoplastic scrotum, cryptorchidism, congenital heart defect, supraventricular fascicular tachycardia, left inguinal hernia, mild muscular hypotonia	Developmental delay, intellectual disability, delayed growth (6 y)	Hu et al. [11] Case 3
				Array	47,XX,+12 (40%)			
20	F	Neonate	Peripheral blood	Chromosome analysis	46,XX	Premature, dysmorphic facial features, short neck, hyperopia with astigmatism, coloboma of left eye, short sternum, widely spaced nipples, pigmentary dysplasia, preaxial polysyndactyly left foot, complete syndactyly of second and third toes, congenital heart defect, hypoglycemia, gastroesophageal reflux disease, mild left hydronephrosis	Normal development (3.5 y)	Hu et al. [11] Case 4
				Array	47,XX,+ 12 (20%)			
				FISH	47,XX,+12 (40%)			
21	F	Neonate	Peripheral blood	Chromosome analysis (2 analyses)	46,XX	Dysmorphic facial features, short neck, single transverse palmar crease on both sides, camptodactyly, clinodactyly digitus pedis 4 on both sides, overlapping toes, deep plantar crease on both sides, anteriorly placed anus, congenital heart defect, tracheomalacia, transient hypopituitarism (with hypoglycemia and hypocortisolism), coloboma, tapetoretinal abnormalities, mild cerebral atrophy, delay of cerebral myelination	Developmental delay, delayed growth, breathing aid, feeding problems, muscular hypotonia, taptoretinal abnormalities, coloboma (13 mon)	Current case
				FISH (2 analyses) FISH	46,XX 47,XX,+12 (3.5%)			
			Urinary cells		47,XX,+12 (28%)			

*ASD* atrial septal defect, *F* female, *M* male, *Array-CGH* array-comparative genomic hybridization, *FISH* fluorescence in situ hybridization

**Fig. 1:**
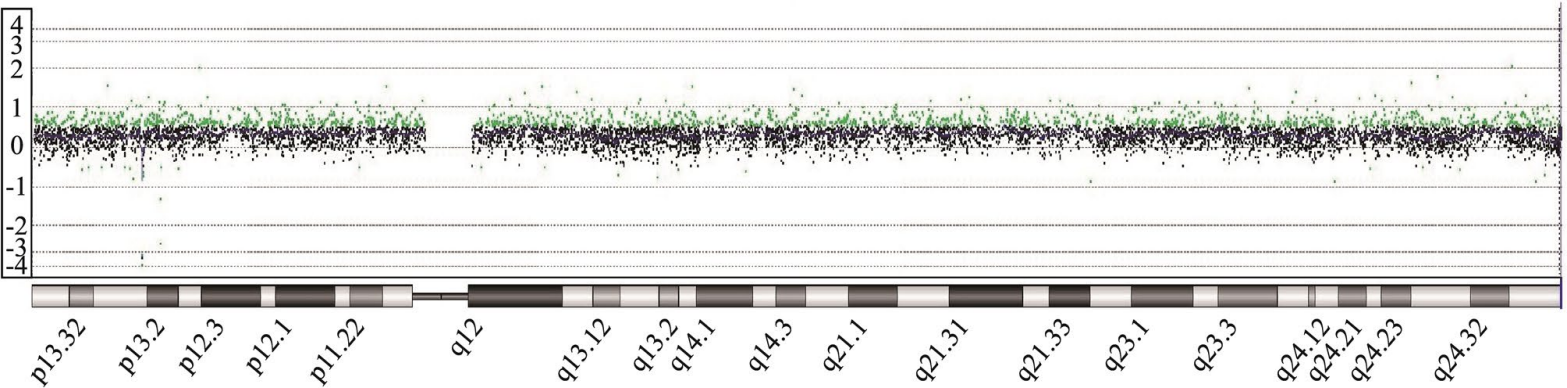
Detailed view profile (array-CGH) of chromosome 12 showing the trisomy 12 mosaicism. X-axis, chromosome 12 ideogram from p (left side) to q arm (right side); Y-axis, intensity

The described patient is the second child of a 37-year-old mother. The healthy parents already had a healthy son. Upon prenatal screening, amniocentesis was performed owing to fetal abnormalities including muscular ventricular septal defect, aberrant right subclavian artery and unbalanced ventricles with hypertrophy of the right ventricle detected by ultrasound examination at gestational week 21. Chromosome analysis revealed in 18/55 metaphases (33%) mosaicism for trisomy 12 in cultured amniocytes. Fluorescence in situ hybridization (FISH) analysis on 58 uncultured amniocytes found 24 cells with trisomy 12 consistent with 41% mosaicism for trisomy 12. An array-CGH analysis (array-comparative genomic hybridization) of fetal DNA displayed an additional chromosome 12 in 30–40% of all analyzed cells (Fig. 1). The parents decided to continue the pregnancy. Ultrasound in gestational week 24 confirmed the abnormalities listed above and additionally showed an increased vascular resistance of the left uterine artery. In gestational week 28, the development of polyhydramnios was discovered. The following ultrasound examinations showed no deterioration of the previously stated findings.

**Fig. 2:**
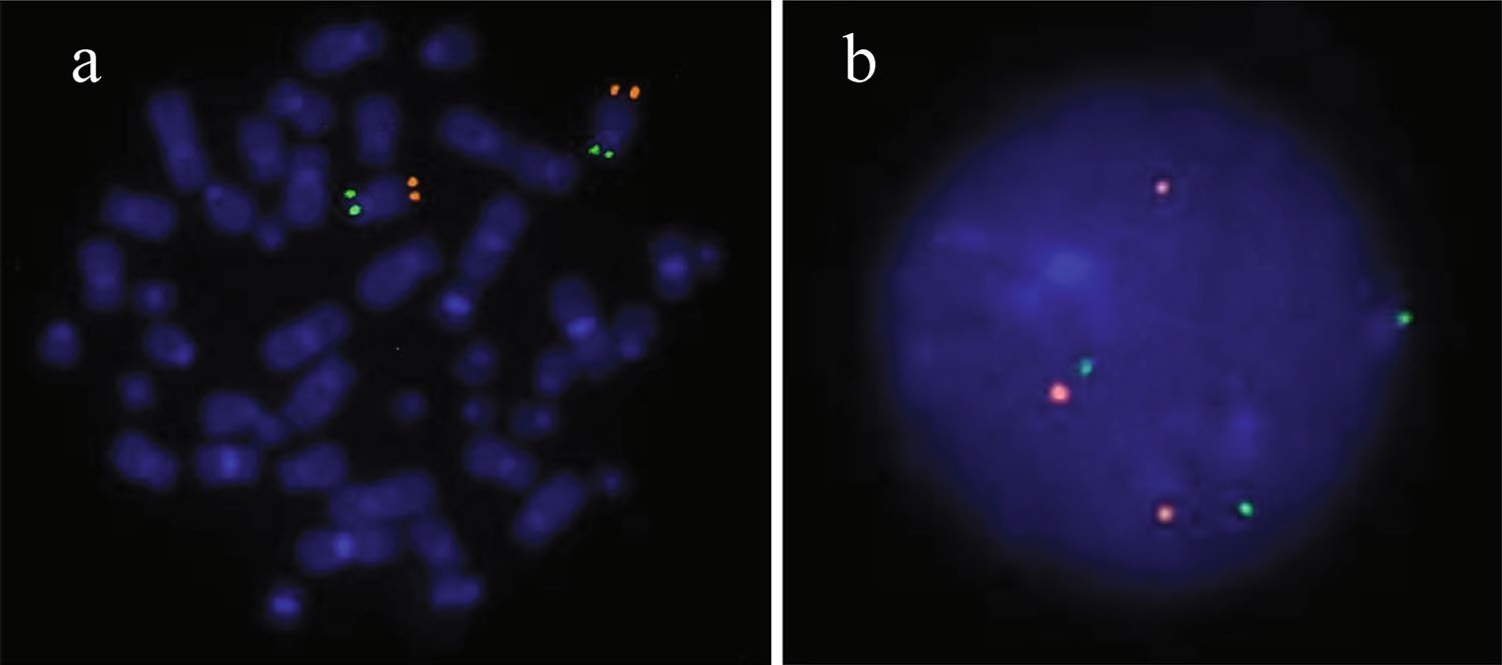
Fluorescence in situ hybridization images of metaphase (**a**) and interphase (**b**) of chromosome 12p subtelomeres (spectrum orange: 12q; spectrum green: 12p; both from Abbott) of lymphocytes showing a regular karyotype (**a**) and trisomy 12 (**b**)

After birth, cytogenetic analysis of neonatal lymphocytes revealed a 46,XX karyotype. Metaphase and interphase FISH analysis on 10 metaphases and 200 interphase cells did not give evidence for trisomy 12. Following these results, interphase FISH analysis on uncultured urinary cells was conducted at five weeks of age and revealed 28% (28/100 cells) mosaicism for trisomy 12. Thereafter, interphase FISH on lymphocytes was repeated showing three signals in 7/200 cells consistent with 3.5% mosaicism for trisomy 12 (Fig. 2). Repetitive chromosome analysis confirmed the normal karyotype of the neonatal blood sample. The varying results described above are shown in Table 2.

**Table 2:** Cytogenetic and molecular genetic findings

Time of sample collection	Tissue	Genetic testing	Karyotype (percentage of trisomic cells)
Gestational week 21	Cultured amniocytes	Chromosome analysis	47,XX,+12 (33%)
	Uncultured amniocytes	Interphase FISH	47,XX,+12 (29%)
	Uncultured amniocytes	Array-CGH	47,XX,+12 (40%)
Neonate	Blood	Chromosome analysis	46,XX
	Blood	Interphase and metaphase FISH	46,XX
5 weeks of age	Uncultured urinary cells	Interphase FISH	47,XX,+12 (28%)
8 weeks of age	Blood	Interphase FISH	47,XX,+12 (3.5%)
	Blood	Chromosome analysis	46,XX

*Array-CGH* array-comparative genomic hybridization, *FISH* fluorescence in situ hybridization

**Fig. 3:**
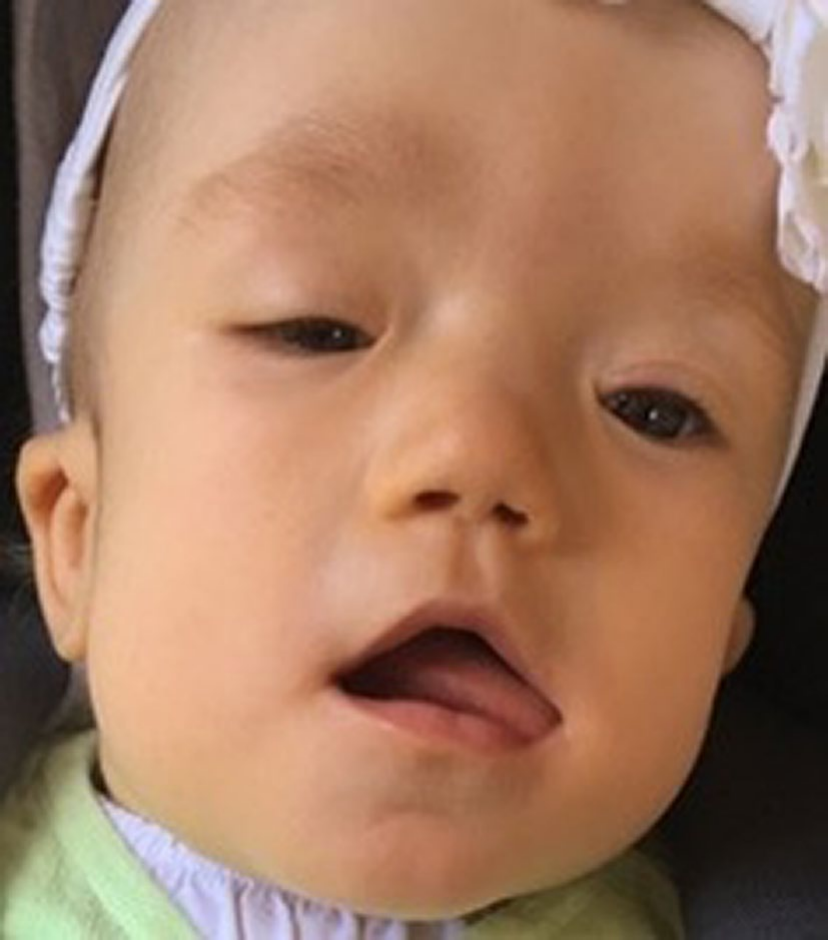
Facial dysmorphic features include prominent forehead, ptosis, epicanthus, left missing upper eyelid crease, broad flat nasal bridge, low-set ears and prominent cheeks. Parents gave consent for publishing the photograph of the case

The female baby was delivered spontaneously at 38 weeks of gestation with a birth weight of 3820 g (91th percentile), length of 52 cm (72th percentile), and head circumference of 38 cm (> 99th percentile). She showed multiple dysmorphic features, such as the prominent forehead, broad flat nasal bridge, low-set ears, prominent cheeks, flat profile, single transverse palmar crease on both sides, camptodactyly of the fifth finger on both sides, clinodactyly of the fourth finger on both sides, overlapping toes, deep plantar crease on both sides, short neck, and anteriorly placed anus (Fig. 3). The ophthalmologic examination displayed a missing upper eyelid crease and coloboma of the right eye as well as blepharophimosis, ptosis, epicanthus, and tapetoretinal abnormalities on both sides. Echocardiography demonstrated a ventricular septal defect, an atrial septal defect, a patent ductus arteriosus botalli, an aberrant right subclavian artery, and a bicuspid aortic valve. At six weeks of age, the child underwent an interventional occlusion of the patent ductus. A tracheobronchoscopy that was performed, after weaning from the respirator failed and after recurrent pneumonias at 10 weeks of age revealed severe tracheobronchomalacia. Additionally, the girl had unexplained episodes of hypoglycemia. Further diagnostics showed a transient hypopituitarism with a decreased level of cortisol. Magnetic resonance tomography (MRI) at 4 months of age detected mild cerebral atrophy with enlarged inner and outer cerebrospinal fluid spaces as well as the delay of myelination in the cerebrum. Owing to feeding problems, a percutaneous endoscopic gastrostomy tube (PEG) was inserted at an age of almost 5 months. The girl stayed in the hospital for about 5 months and was discharged with a non-invasive respiratory support and PEG. She was breastfed and ventilated during the day only with continuous positive airway pressure. At night, the girl was fed over the tube and was ventilated with a pressure-controlled ventilation. Five months later, she did not need respiratory support during the day anymore. By 13 months of age, the girl still had pressure-controlled ventilation overnight. She was breastfed, ate baby foods, and was given only water via the PEG. Her weight was 8100 g (8th percentile); her length was 71 cm (3rd percentile); and her head circumference was 46.5 cm (66th percentile). The brainstem evoked response audiometry displayed a normal signal in the left ear and could not be analyzed in the right ear due to a narrow ear canal. The pupillary light reflex showed no abnormalities, and she was able to fixate briefly on an object and people. She presented with an unremarkable smooth pursuit eye movement but with a persistent nystagmus. Coloboma of the right eye and tapetoretinal abnormalities were still present. Closure of the atrial septal defect is scheduled. Concerning the terms of development, she reached the age-appropriate milestones with delay. At 9 months of age the girl was able to roll to the sides and from front to back, but not from back to front and could lift and uphold her head for a few minutes. At 13 months, she could raise her chest supported by arms when placed in the prone position for a short period of time. She was still not able to roll from back to front. She transferred objects from hand to hand and sometimes used a pincer grip but had general muscular hypotonia. She was babbling but not using any words. She received physical and occupational therapy. According to the new classification of genetic mosaicism the mosaicism status of the individual can be classified as follows: A3B2C1D4aE1F3 [9].

The phenotype of trisomy 12 mosaicism as reported in the literature is variable and, therefore, recognition is quite difficult. Consistent abnormalities that have been found in at least three of the up to now reported patients are dysmorphic (cranio-) facial features, developmental delay, intellectual disability, pigmentary dysplasia, congenital heart defects, muscular hypotonia, microcephaly, short neck, and short stature (Table 1). Of these shared features, our patient had dysmorphic facial anomalies, a complex congenital heart defect, developmental delay, and short neck. Parallels may be drawn to Leschot et al. and Hu et al. (case 4), whose patients also had unexplained hypoglycemia [10, 11]. Further diagnostics in our patient showed a transient hypopituitarism with a decreased production of cortisol. Another similarity can be discovered with the patient reported by DeLozier-Blanchet et al. who had camptodactyly as well as with the patient described by Al-Hertani et al. who also suffered from tracheomalacia [4, 12]. However, the latter was induced by a vascular ring, in contrast to our patient. Hu et al. also described an anteriorly placed anus in one patient (case 1), cerebral atrophy as well as enlargement of the lateral ventricles (case 2), and a coloboma of one eye (case 4) [11]. These features occurred in our patient as well. Varying dysmorphic (cranio-) facial features appear to be the most common finding occurring in 16 of the 21 cases followed by congenital heart defects detected in nine cases (Table 1).

Furthermore, using the Face2Gene application might be an additional possibility to help support the prenatal diagnosis of mosaic trisomy 12. The Face2Gene application is a facial analysis technology using a software called DeepGestalt. It provides valuable assistance for recognizing genetic syndromes by analyzing facial images of patients [13]. Because phenotype descriptions are rather subjective and the phenotype of trisomy 12 mosaicism is quite variable, which complicates its definition, using an automated facial analysis might lead to a faster diagnosis.

Providing a prognosis for children with mosaic trisomy 12 might be challenging because there seems to be no association between the degree of mosaicism and the severity of the phenotype. Von Koskull et al. reported a girl with 25% mosaicism for trisomy 12 in skin fibroblasts with a complex congenital heart defect who died at five weeks of age after an attempted surgical heart procedure [14]. Bischoff et al. described a rather low incidence of trisomic cells (5%) in spleen tissue in their patient who had an unfavorable diagnosis of Potter sequence. The patient died immediately after birth [15]. The same percentage of trisomic cells was found in a female patient described by Chen et al. with no phenotypic abnormalities at all [1]. Boulard et al. reported a 15-year old girl with 80% mosaicism for trisomy 12 in ovarian fibroblasts. She presented with pituitary stalk interruption, polycystic ovary syndrome, strabismus, conductive hearing loss, atrial septal defect, and delayed growth but normal cognitive development [16]. In the four cases with a fatal outcome, the detected counts for mosaic trisomy 12 ranged from 5 to 26%. The percentage of trisomic cells in the other 17 patients varied from 3.5% to100% (Table 1). These observations give no evidence for a correlation between a lower mosaic trisomy 12 level and a less severe outcome, nor a higher mosaic trisomy 12 level and a more severe outcome. Table 1 also illustrates that no clear association exists between the manifestation of clinical features and the type of tissue trisomy 12 cells.

Moreover, there have been observations regarding differing results in repeated amniocentesis complicating genetic counseling already prenatally [1, 17]. Inconsistent trisomy 12 mosaicism levels have been reported by various authors. For examples, Spiro et al. reported 7.5% versus 48%, Chen et al. reported 16.7% vs. 39.1% in 2013 and 17.2% vs. 40% in 2017 [1, 17]. These findings make a reliable prognosis almost impossible. Additionally, as Aughton et al. and Al-Hertani et al. already have stated, there seems to be a female preponderance with mosaic trisomy 12 [4, 18]. Table 1 shows that the male:female sex ratio of patients diagnosed postnatally is now 4:17 supporting the statement above. The reason for this phenomenon is thus far unknown.

Cytogenetic studies in the blood are an efficient method for the detection of mosaics, especially if an adequate number of cells are analyzed. However, this method may not be sufficient or decisive if there are mosaics restricted to tissues. The present case confirms again that genetic analysis of the blood can be an unreliable indicator of the child’s karyotype after prenatal detection of mosaic trisomy 12. In our patient, trisomic cells were detected in only one of four blood analyses and ultimately with a rather low incidence of 3.5% compared to the prenatal results. This might be due to the fact that the amniotic fluid contains cells of all three germ layers (endoderm, mesoderm, ectoderm), and analysis of blood lymphocytes only represents cells of the mesoderm. Hu et al. reported similar results with mosaic trisomy 12 detected in only one of four blood analyses as well (case 1) [11]. These results are consistent with several other cases in which trisomic cells were not found in the blood at all but were found in different tissues. In our patient, the presence of mosaic trisomy 12 could be confirmed for the first time in urinary cells after birth. Out of the 21 cases, trisomic cells were detected in peripheral or cord blood in 41% analyses (14/34), in skin cells in 59% analyses (10/17), in urinary cells in 83% analyses (5/6) and in 100% analyses in ovarian cells (1/1), spleen cells (1/1), and buccal cells (1/1). In addition, this review of the literature suggests that identifying mosaic trisomy 12 is not always achieved using chromosome analysis. Chromosome analysis was performed 42 times but only detected trisomic cells 17 times. Array-CGH identified trisomy 12 mosaicism in four out of five conducted analyses, FISH in eleven out of 13 analyses. Thus, chromosome analysis led in 40% to a correct diagnosis, whereas FISH detected trisomic cells in 85% and array-CGH in 80%.

In conclusion, sole analysis of peripheral or cord blood, as well as using only chromosome analysis for diagnostic testing, might fail to reveal mosaic trisomy 12. Once again, it is evident that classical cytogenetic techniques are still the gold standard for the detection of low-level mosaicism and chromosomal rearrangements. Chromosomal microarray analysis cannot detect this kind of chromosomal aberration owing to its limitation (> 20% mosaic cell lines). However, in cases in which it is found prenatally or if a constellation of abnormalities including dysmorphic facial features, congenital heart defects, pigmentary dysplasia, hypotonia, and developmental delay is detected postnatally, several tissues should be analyzed for identification of mosaic trisomy 12. In addition, it should be taken into account that FISH and array-CGH allow the examination of more cells than chromosome analysis along with a higher chance to detect the mosaic trisomy 12. Therefore, if mosaic trisomy 12 is not revealed by chromosome analysis, FISH or array-CGH should be done as well. It should even be considered to perform FISH or array-CGH first.
